# Abstracts and Gleanings

**Published:** 1880-07-20

**Authors:** 


**Local Anæsthesia with Bromide of Ethyl.**—M. Terrillon stated at the Societe de Chirurgie that he had employed the bromide of ethyl about a dozen times in operations with the thermo-cautery. In a minute or two a white patch indicating cutaneous anæsthesia is produced, and on the pulverization being continued, insensibility of the tissues is produced to the depth of two centimeters. The production of the white patch is not essential, as anæsthesia may exist when it is absent. The results have proved very satisfactory, but in two cases M. Terrillon did not succeed, owing, as he believes, to the pulverizators which he employed having too small a jet.—*Gaz. Med.*

### ABSTRACTS AND GLEANINGS.

**Sulphur and its Compounds in Skin Diseases.**—Dr. L. Duncan Bulkley, of New York, in the American Medical Association, presented a paper on this subject. He referred to the great popularity which sulphur had had in the treatment of skin diseases, and of the indiscriminateness of its employment. His present aim was to show in exactly what diseases sulphur really relieved, and how it should be administered.

First, as to its internal use. *Pure sulphur* was seldom given alone in skin diseases. In eczema about the anus and genitals, however, it is very useful, especially if there is any constipation or piles. It may be given with equal parts of cream of tartar, in teaspoonful doses. Sulphurous acid is rarely used internally.

*Sulphide of calcium* is very valuable in skin lesions attended with suppuration. In acne it is often useful, but chiefly in those cases which have a considerable pustular element. It is not of much use in acne rosacea. In hordeoleum it is very valuable; also in furunculosis, relieving not only the symptoms, but preventing further crops of boils. Like testimony may be given regarding its effects in carbuncle and suppurating buboes. True, non-parasitic sycosis is sometimes benefited by sulphide of calcium. The drug is liable to be poor, and should have its characteristic smell of sulphuretted hydrogen. Dr. Bulkley usually gave gr. 1/4 q. i. d.

*Sulphuret of potassa* probably has the same effect as the sulphide of calcium.

It is undoubtedly the sulphur that does the good in these cases, for other combinations of sulphur, such as the hypophosphite and sulphuric acid, have been found similarly beneficial. A wonderfully valuable combination of sulphur is that known as "Startin's Mixture":

℞. Magnes. sulph................℥j.

Ferri sulph...................ʒj.

Acid sulphur. dil.............ʒij.

Tr. gentian...................℥j.

Aquæ..........................℥iij

M. Sig.—℥j dose after meals.

This is very potent in reducing cutaneous congestion in such conditions as erythema multiforme, erythematous eczema and urticaria.

In regard to the use of natural sulphur waters, some benefit is obtained from them, but it is impossible to speak definitely of them until more data are collected. The speaker wished help from any in collecting such facts.

Externally, sulphur has gained its widest reputation in the treatment of scabies, for which it is almost a specific. It should be remembered that sulphur is an irritant to the skin.

It is also beneficial in acne, either in the form of the pure sulphur or the hypochloride, the latter being used as an ointment, about ʒj to ℥j. Sulphur will also destroy the parasites of favus, ringworm and tinea versicola—pure sulphurous acid being the best form for these.

Sulphur vapor baths are of value in very few diseases of the skin. They stimulate the skin and liver, and they destroy skin parasites; but not much more can be said for them.

**Jamaica Dogwood.**—A writer in the Georgia Eclectic Medical Journal says of this new remedy:

In toothache it is an absolute specific where a cavity exists. Saturate some loose cotton with the tincture and insert it in the cavity and the pain will at once vanish. I have treated several hundred cases in this manner without a single failure.

I have experimented upon myself and the following are the provings: I commenced with small and repeated doses until I got the full physiological effect. The first sensation is a fullness and warmth through the chest, strong heart beat and full pulse; this is followed by great internal heat which slowly extends to the surface and is followed by perspiration. Should the medicine be still further continued, a violent headache is the result which passes off in a few hours. It has been proposed to substitute it for opium as it leaves no bad effects, whilst the latter disagrees with a large number and in all cases deranges digestion. I find that Jamaica dogwood is both an anodyne and a narcotic, but it is dificult to measure the dose to produce this result. Enough must be taken to produce diaphoresis and sleep, but then it becomes necessary to stop the administration, otherwise headache will result. I usually prescribe:

℞

F. Ex. Jamaica Dogwood.......................... ℥ i.

Elixir Simplicis........................................ ℥ iii.

M.

Sig. A teaspoonful every half hour until perspiration ensues.

I have used it with great benefit in angina pectoris and combined with yerba in the cough of phthisis.

In intermittent fever I find it answers the purpose as well as quinine, abating the paroxysm in a majority of cases.

In sciatica I find it of the greatest benefit. In six cases that I have had under my care I succeeded in effecting a cure by the internal administration of Jamaica dogwood and the external application of mullien leaves steeped in warm vinegar. Nerve stretching and the hypodermic injection of morphia and chloroform will now be of the past.

It will be my first treatment in acute rheumatism, and judging from analogy, ought to be successful.

I trust that others will investigate this drug and give us the benefit of their experience.

**Oxalate of Cerium in Coughs.**—Dr. Morje, in a report upon the use of this article in St. Luke's Hospital, says:

The wards were evidently quieter while those requiring cough medicines were taking the oxalate than at other times. The remedy was on several occasions entirely intermitted in the wards for a few days, and then resumed as each case came to require it. In about twenty-four or forty-eight hours after stopping it the noise and complaining would increase.

The best effects were noticed in the early stages of phthisis, or in chronic bronchitis; but a hoarse, harassing cough, with profuse expectoration in the third stage, was in several cases relieved, the cavities being then emptied without violent coughing or great effort.

The drug was not thoroughly tested in acute cough, but my impression is that it is less efficient in acute than in chronic cough, whether pulmonary or laryngeal. Nausea and vomiting became rare among the phthisis patients during the time it was used. It never disturbed the stomach, but, on the contrary, appetite and digestion often were speedily improved.

In dyspnœa having an asthmatic element, it doubtless affords more or less relief. In dyspnœa due to loss of normal lung substance, it can give the relief only which is procured by checking the coughing; during coughing the legitimate functions of the still healthy portions of the lungs are more or less arrested.

Frequently sleep was restored, and occasionally considerable drowsiness attended its use for a time, but these effects were not attributed to any narcotic property of the drug, but to the relief of the cough, which allowed the patient to make up for previous loss of sleep.

The patients taking the oxalate were weighed weekly, and several very soon showed marked gain.

No dangerous symptoms were ever observed attributable to the drug. The largest dose given was twenty grains. This dose was never repeated the same day, but was given, in a few instances, to patients taking 10 grains twice at other periods of the day. No complaint was ever voluntarily made of its causing dryness of the mouth, but several of the patients questioned on this point admitted that it produced this symptom at first, but declared that after taking it a few days they ceased to notice it. There were now and then complaints of its producing a sensation of tightness in the chest, and of its drying up the expectoration, but complaining of these symptoms was common when the oxalate was not being used.

**The Opium Habit—A Possible Antidote.**—The Louisville Medical News, May 29, contains an admirable paper from the pen of Prof. E. R. Palmer, on "The Opium Habit—a Possible Antidote."

This paper begins with a reference to the great physical distress and pernicious influence induced by the opium habit. Reference is next made to the various claims of remedies as a curative of this habit, which are disposed of in the following words: "If any man has yet discovered a cure for opium eating, I am sure the medical world is not aware of it."

Prof. Palmer goes on to say that recent experience has led him to hope that he has discovered a cure in the fluid ext. of coca, made by Parke, Davis & Co. This drug Prof. Palmer employed in practice with such results as satisfied him that it possessed uncommon merit as a remedy in the treatment of the opium habit.

He says, after relating several cases in which he employed this agent: "These are very brief and slender claims upon which to base a claim of discovery; and while I might supplement them by several cases of ordinary hypochondriasis relieved by the agent in question, I do not deem it worth while, as my only desire is to direct professional attention to the administration of coca in the treatment of the opium habit.

Erythroxylon coca is a native of the eastern slope of the Andes. It is cultivated in the tropical valleys of Bolivia and Peru. The greatest of care is given to its culture by the natives. An idea of its importance as an agricultural product may be gained from the fact that the duties upon coca in Peru amount yearly to four hundred thousand dollars. The Peruvians are preeminently a despondent, an unhappy race, and coca is their balm. To them it is a relic of departed days of glory, and under its benign influence they enjoy in dream and delirium the halcyon days of Monco Capac.

Prof. Steele, of the American Pharmaceutical Association, from whose article on coca I glean these facts, says: 'Coca is both salutary and nutritious; in fact, the best gift the Creator could have bestowed upon the unfortunate Indians. They always carry a bag of leaves suspended from their necks, upon which they draw three times a day with as much pleasure and delight as a connoisseur in tobacco smokes a fragrant Havana. It imparts brilliancy to the eye and a more animated expression to the features, agility to the step, and a general appearance of animation and content.' Indeed, one can scarcely read Prof. Steele's article without wishing to test the virtues of this great antidote for the blues. The ordinary dose for adults of the fluid extract is a tablespoonful."

The opium habit is a most unfortunate condition, and anything offered for its relief should be carefully tested. Prof. Palmer's experience with the use of coca goes to show that if not a positive cure in every case, it merits professional attention as an agent of great value. —*Md. Med. Jour.*

**Arsenic in Chorea.**—According to Bouchardat's Ann. for 1880, we find that, from observations made in the service of M. Siredey, by M. Pomel, upon the treatment of chorea, the following deductions are made:

1st. Of all the remedies employed in chorea, those which are the most rapid and sure are the arsenical preparations; particularly arsenious acid, which at once produces rapid amelioration of the symptoms, and brings about a speedy cure.

2d. Grave cases of chorea that have resisted other treatment yield, frequently with promptness, to arsenic.

3d. To obtain the full benefit of the arsenical treatment, it is necessary to administer the medicine in such doses as to produce, as speedily as possible, the constitutional effects or signs of arsenical saturation.

4th. Even in children affected with chorea, no hesitation should be felt in giving strong doses of arsenic, in order to reach the point of saturation quickly.

5th. Without denying the possibility of danger in the use of arsenic in the treatment of chorea, yet no case has thus far been recorded to establish the fact.

The preparation used by M. Siredey is Boudin's solution, containing 1 milligram (1-65th of a grain) of arsenious acid to 1 gram (15 grains) of solvent.

M. Siredey commences, in the case of a young and moderately vigorous subject, with ten grams of the solution a day, well diluted, and increases the dose each day by five grams. This dose is first diluted and then taken in divided doses during the day. Should vomiting or nausea occur the quantity must be diminished. For a child of eight or ten years, from two to four grams of the solution will be sufficient to commence with, increasing it daily by two grams.

*Note by Translator.*—As the solvent in Boudin's preparation is not given, Fowler's solution may be substituted. Its equivalent dose would be twenty minims daily, increased in the above proportion.—*Med. and Sur. Rep.*

**Strong Calvanic Current in the Treatment of Sciatica.**—Dr. V. P. Gibney, of New York, read a paper on this subject. In 32 cases thus treated at the Hospital for the Ruptured and Crippled, 24 were entirely relieved, 3 moderately relieved and 5 not relieved. The currents were given daily; sixteen of the cases had had no relapses at date; several others had only slight relapses, and only four had a permanent return. Several cases were related. In one, twenty-seven cells were applied for ten minutes daily for several days, with rapid relief. The duration of the disease in the cases reported varied from a few weeks to several months.

The current should be a stabile one; the labile current is not a constant one. The speaker described the best form of battery. The current should be just as strong as the patient can bear it. The application should be given for ten minutes, or even fifteen, if possible. It should be given twice a day at first, if possible, and kept up for fifteen or twenty days. If by that time no good results ensued, it had better be discontinued. Six to ten seances may secure success. The descending current is preferable.

Dr. A. D. Rockwell, of New York, said that electricity in any form will not always cure sciatica. He cited cases cured by the faradic current.

Dr. L. D. Bulkley, of New York, referred to two cases relieved by the strong galvanic current.

Dr. Geo. M. Beard, of New York, spoke of the relative value of strong and weak currents. In whatever way electricity may be given, it will sometimes cure neuralgia. Practically, he did not find much difference in results whichever way he applied the current—although, on the whole, the positive pole is somewhat soothing, and the negative irritating. He thought that there was much value in the treatment suggested, but there was a caution needed, for he had seen cases injured by over-doses of electricity. He would never begin a treatment of sciatica with such powerful currents, but would use weaker ones at first.

Dr. Caldwell, of Baltimore, remarked that this is the largest nerve trunk outside of the bony cavities; hence, many cases of neuralgia are due to mechanical interferences, enlargements, tumors and other pressures. When no form of electricity will avail except to diagnose—and here the surgeon is the proper party to relieve this painful malady—it would be unscientific to continue electricity and palliatives indefinitely.—*Proc. Amer. Med. Asso.*

**Hypodermic Injection of Ether in Sciatica.**—I have encountered several cases of sciatica in which the usual treatment of quinine, aconite, colchicum, iodide of potassium, counter-irritation and hypodermic injections of morphia, either produced no results at all, or only temporary results. The disease would sometimes be allayed for a short time and would then manifest itself with as much or with more violence than before. The following is my experience with the hypodermic use of ether:

CASE 1. A large, robust man, aged forty-five years, had been subject to sciatica every spring. April 23, 1879, I introduced twenty minims of sulphuric ether, throwing it deeply into the tissues of the thigh, as near as possible to the seat of pain. The injection was followed by considerable pain, which in a few minutes subsided, and marked relief from the pain of the sciatica was also experienced. On the following day, finding him much better, but not entirely relieved, I repeated the injection, using thirty minims instead of twenty. On the third day he was on the street, and although his pain previous to this treatment had been excruciating, he has for many months been free from any trouble with the sciatic nerve.

CASE 2. At about the same time a neighboring doctor employed the same treatment for the relief of a male patient who had been suffering from sciatica. In this case, as in the other, pure ether was introduced without resulting inflammation or abscess.

Hereafter my practice will be to introduce twenty or thirty minims, directly over the seat of pain, or as near it as possible, and at once to apply to the part a mixture of one part of carbolic acid to two parts of glycerine. The object of this application is the prevention of possible inflammation and abscess.—DR. W. T. SPEAKER in *Chic. Med. Jour.*

**Nausea of Pregnancy—Flatulent Dyspepsia.**—Dr. Norwood, in a paper before the Lancaster County Medical Society, and published in the Medical and Surgical Reporter, July 10, 1880, says the following formula is very efficacious in nausea of pregnancy, as well as useful in flatulent dyspepsia, for which it was first suggested by Dr. Wood, U. S. D.

To make the prescription clear to the eye of the reader at a glance, we place it in regular form, as follows:

℞. Rad. columbo contus.,

Rad. zingiber.. " aa...... ℥ss

Fol. sennæ, ................. ʒj

Aqua bullient., ............. Oj

Infus. M.

SIG.—Take a wineglassful before each meal.

In some cases, attended with unusual acidity of stomach, we add half a drachm of carbonate of magnesia to the recipe. Constipation of the bowels nearly always exists in this sickness; but should the bowels, on the contrary, be relaxed, we omit the senna.

We are in the habit of prescribing two parcels of the recipe for the patient to take continuously, mixing one at a time, and making the addition of water referred. This quantity is sufficient in mild cases; of course it must be continued longer if necessary. There appears a tendency on the part of the complaint to return in the course of three or four weeks after the conclusion of the first treatment. By then using one or two pints more of the infusion, the patient will be almost invariably cured of her sick stomach.

When the complaint is at all violent, it is better for the patient to take the morning dose, and afterward her breakfast, in bed, and not to rise until at least one hour after eating.

Patients rarely present themselves for treatment until they are very much exhausted from this sickness. They are weak, pale or sallow, emaciated, constipated, nervous, and express themselves as feeling wretched generally. In the columbo and ginger we have the tonic and stomachic so much needed, and in the senna the gentle laxative; all of which being combined, mildly stimulates to healthy action the disordered stomach, liver, kidneys and bowels. The bitter taste of the medicine, so objectionable at first to the patient, already so extremely nauseated, soon disappears upon the establishment of healthy digestion and return of natural appetite. The morbid mental condition of the patient clears up and disappears, as does the pale and sallow complexion.

We have been in the regular practice of administering this infusion to all cases of 'morning sickness' that have come under our charge during the last twelve years, numbering perhaps two hundred or more, and such has been our uniform success that we will remark, in closing, though the expression may appear extravagant, that we regard the columbo, ginger and senna infusion as much entitled to the character of a specific in the treatment of the sick stomach of pregnancy, as quinine is in the treatment of intermittent fever.

**Removal of the Uterus by the Extirpation of Tumors Connected with that Organ** was the title of a paper by Dr. T. Gaillard Thomas, of New York. There were three circumstances under which complete extirpation of the uterus might now be regarded as a legitimate, and often a very necessary procedure. 1. Malignant disease. 2. As an addendum to the Cæsarian section, after the method of Porro. And, 3, to render practicable the removal of tumors which took their origin in its tissues, or which arose in the ovaries, and whose attachments were too firm to be broken. It was with the third class that the present paper was concerned, which embodied the results of seven cases, in one of which the whole fundus, in another the whole body, and in five of which the entire uterus was removed. Four of the tumors demanding the operation were large, solid fibroids, with no cystic elements. One was a fibro-cyst, partly solid and partly fluid, and one a peculiar ovarian tumor which, developing between the layers of the broad ligaments, lifted the uterus entirely out of the pelvis, and made it a mere addendum to their walls. Out of the seven cases four recovered and three died. The three fatal cases were all operated on for large, solid tumors. Of the four successful ones, one was a case of solid uterine fibroid, one a case of large fibro-cyst, and two cases of ovarian cysts with large amounts of solid material in their walls. On recognizing this fact, it was to be borne in mind that a tumor susceptible of diminution of size by tapping was not so dangerous for laparotomy as one which, being entirely solid, involved the necessity of a long abdominal incision. Dr. Gilman Kimball, of Massachusetts, has removed the uterus thirteen times (nine times for solid and four times for fibro-cystic tumors), with the excellent result of eight recoveries and five deaths. In some of his cases the whole, in others a part only, of the uterus was removed.—*Trans Amer. Med. Asso.*

**Introduction of Liquids into Eustachian Tube and Middle Ear,** Was the title of a paper by Dr. S. S. Jones, of Chicago. The attempts of Sir Astley Cooper, in 1800, by puncturing the membrane, and pushing air through the external meatus and Eustachian tube, and, later, of Horner, of Philadelphia, of Hinton and Allen, of London, induced the author to resort to one or the other means of introduction into the tube—that is by the nares or external ear. He has used liquids in this way for nine years, and is convinced of its advantage in dry, chronic, non-suppurative inflammation of the middle ear. Dr. Jones reverses Horner's method of procedure, and introduces liquids through the Eustachian catheter into the tube. The tube is thus more easily dilated, and will finally better admit air. He has abandoned bougies as dilators. He argues from the benefit derived by proper applications to the post-nasal cavity and pharyngeal vault a similar benefit from the local contact of fluids of proper temperature to the Eustachian tube or cavity of the middle ear. The bad influence of sea-bathing upon the middle ear has been over-estimated. As naval surgeon in the war of 1861–65, he noticed less acute inflammation of the middle ear when the temperature allowed sea-bathing on the part of the sailors, than when the thermometer was so low as to prevent it. Dr. Jones advised slightly saline tubal injections on account of their better tolerance than pure water, or weak solutions of borax, chlorate of potash, etc. Its temperature showed 60° at blood heat; no force should be used, and the quantity should be regulated by the amount of effect desired. The comfort of the patient, and the subsequent improvement in hearing operate as warrants for the continuance of the practice.—*Tr. Amer. Med. Asso.*

**Phosphate of Bismuth.**—M. Tedenat recommends, in Montpellier Medical, the employment of phosphate of bismuth in preference to the subnitrate. The anti-diarrhœal action of phosphate of bismuth is manifested in the same way as the subnitrate. In consequence, however, of its greater insolubility, the phosphate acts in rather weaker doses, especially in gastric affections. Notwithstanding the acidity of the gastric fluids, it is not in the slightest degree affected, which is but natural, since it resists strong acids, even in a concentrated form. The phosphate acts in somewhat smaller doses than the subnitrate, and the difference in activity is sufficiently great to create a superiority, from this point of view, in favor of the phosphate. The doses are likewise varied, according to the nature of the case. They are usually about from one to two grams. The mode of administration is absolutely identical with that of the subnitrate. With infants it suffices to place the desired quantity on the tongue, and to offer the breast or bottle. The salt is easily conveyed into the stomach, and it is possible by this means to administer large doses. Adults take this drug suspended in some liquid. In many cases some advantage is found in making it into sweetmeats and pastilles of about one or two grams. They become disintegrated in the mouth, and the phosphate is gradually conveyed into the stomach without the patient having been able to perceive the presence of an insoluble salt in the mouth.—*Med. and Sur. Reporter.*

**Successful Treatment of Imperforate Anus.** — Sunday, December 1st, 1879, says Dr. Geo. W. King, in Mich. Med. News, I was called to see a child a few hours old, the midwife in attendance having discovered something wrong with it. Upon examination I found it to be a case of imperforate anus. Perineum was smooth, and without a trace of the anal aperture. The child—a male—was, with this exception, well developed and in good condition, but seemed to be suffering acutely from the intestinal obstruction. The parents insisting that an attempt must be made to relieve the child, I decided to make an exploratory incision to determine the location and condition of the rectum if present. After emptying the bladder by pressure above the pubis, I made an incision of about one inch and a-half in length in middle line, beginning a little in front of coccyx; then by a careful dissection separated the tissues to the depth of one inch, at which point the rectum was found to end in a *cul-de-sac.* The intestines being distended with meconium, I punctured the sac with a trochar, and a free discharge of its contents followed; enlarging the opening by a crucial incision, and clearing the cavity, the next step in the operation consisted in bringing down the bowel and fastening its mucous membrane to the edges of the external wound by means of sutures. This proceeding was accomplished without difficulty; the hemorrhage was free, but easily controlled by torsion and cold compress. The case progressed well, and the union has been rapid. The treatment consisted in keeping the orifice dilated with a plug of oiled cotton, and the introduction of a bougie occasionally.—*Mich. Med. News.*

**Catarrh of the Bladder in Old People.**—The important practical point in relation to treatment is first to ascertain the occasion of the local catarrh. In nine out of ten of these cases it consists in inability, often only to a slight extent, on the part of the patient to empty the bladder completely. The universally acknowledged cause, hypertrophy of the prostate, is, of course, the first in order of frequency. But after this are others not infrequent. Defective action may be due, first, to simple atony, the result of past habitual or occasional over-distension of the bladder with urine; secondly, to thickened and incompetent muscular paralysis of the bladder after chronic inflammation, sometimes associated with old stricture; thirdly, to defective innervation seen in connection with other slight signs of impaired function in a nervous centre, the last being, of course, the most serious of all, in its nature and probable results.

In all of these, local treatment, by carefully removing all the secretion by means of a soft catheter two or three times a day, perhaps aided by gently washing out the remainder, is the chief efficient remedy. Remember that this incompetence of the bladder is always to be sought for by physical examination; no other form of evidence in relation to it, as the patient's sensation, etc., is to be accepted as trustworthy. The introduction of a soft catheter immediately after the patient has passed water by his natural efforts, is the only test, and it should be applied on two or three occasions before arriving at a definite conclusion. The casual relation between the group of symptoms enumerated at the outset, and the defective function described, is far more common than it is generally supposed to be. It is on this account, therefore, that I have asked your attention specially to the subject.—Sir Henry Thompson in *London Lancet.*

**Medical Uses of Carbolic Acid.**—The diseases in which carbolic acid is especially useful are :

1. All that class of local festering, pustulating diseases of the skin which are at once so common and so difficult to cure. They include all kinds of pustules, boils and carbuncle; sycosis, pustular, acne and festering ringworm.
2. Such strumous sores (especially of the neck) as come under the care of the physician.
3. Excoriations of the os and canal of the cervix uteri.
4. Phthisis in its second and third stages, and cases of bronchitis accompanied with more or less purulent expectoration.

In order to be effective the carbolic acid must be brought into contact with the part to be acted on, and in many cases where it has been found ineffective the failure has been due to a neglect to insure this contact. In the pustulating and suppurating diseases of the skin it is never sufficient to apply the solution of the acid, of whatever strength, upon or to the outside of the skin. It must always be introduced into the interior of the sore or pustule itself, and so as to come sufficiently in contact with every part of the diseased surface.

All cases of boils and carbuncles in their earlier stages can be absolutely aborted and cured, whilst even in later stages their further increase can be almost surely prevented. For this purpose a very strong glycerine solution should be employed, and it is best conveyed into the interior of the pustule, boil or suppurating spot by a new quill pen dipped into the solution, and introduced by a rotatory motion through its apex, where a sufficient aperture will generally be found. In carbuncles, which are necessarily larger, and have often several openings, several such introductions may be necessary, or, at a later period, threads of lint soaked in the fluid may be passed with a probe well into all the sieve-like openings. Occasionally, as when the mass is large and solid, a watery solution of the acid may be injected with a hypodermic syringe into various parts of the hardened growth. The same plan of treatment is often quite effective in cases of sycosis, pustular acne and festering ringworm.—Dr. E. P. Eade, in *Lancet.*

**Wickersheimer's Preservative Fluid for Animal Substances,** was the title of a paper by Dr. E. Gruening, of New York. The Prussian government, a few months ago, bought and published the following formula : To 3,000 parts of boiling water put 100 parts of alum, 25 parts of common salt, 12 parts of saltpetre, 60 parts of carb. of potash, 10 parts of arsenious acid. Cool and filter, and add to 10 parts of this solution, 4 parts of glycerine and 1 part of methlyic alcohol. In the Centralblalt f. dei med. Wissenschaften for January, 1880, Dr. Boesicke states that the formula published by the Russian government contains two errors. He obtained a different formula directly from Wickersheimer, in which, instead of 10 parts of arsenious acid and 1 part of methylic alcohol, there should be 20 parts of the acid and 4 parts of methylic alcohol. The government formula is slightly opalescent, while that of Boersicke is perfectly clear. The first preserves the refractive media of the eye, while the second does not. A small, soft eye, which had lain three months in the government fluid, shows the same depth of anterior chamber, a clear cornea and an unchanged blue iris. The glioma retinæ, for which the eye was removed, can still be diagnosed by the naked eye. An albinotic rabbit's eye having laid in the formula the same length of time, shows the media clear, and gives the pink reflex. With the Boersicke mixture preservatton is less perfect; the lens becomes opaque in a few hours, and the cornea dim after a few days, as if it had been placed in absolute alcohol.—*Va. Med. Monthly.*

**Chloral Hydrate in Acute Gastro-Enteritis of Children.**—Prof. Adolphe Kjellberg finds that there is no medicine which is of so much use as chloral in checking the vomiting in acute gastro-enteritis of children. Being rapidly absorbed, it stops the vomiting, calms the patient, and often checks the diarrhœa.

It is best given by enema, so as not to risk its rejection by the irritable stomach. It should be given soon after the bowels have been moved. The dose for a child of from five to six months is twenty-five to thirty centigrams (three and a half to four grains), while to a child of from twelve to fifteen months fifty to sixty centigrams (seven to eight and a half grains), may be given. The bulk of the injection should not exceed a dessertspoonful. The enemata may be repeated two or three times daily, and the dose may be increased if it is found necessary. In order to increase the effect of the chloral the author generally adds to each enema a drop of tinct. opii. and, if stimulants be indicated, five to fifteen drops of liq. Hoffman. At the same time other remedies are not neglected—iced water or cognac or champagne for the vomiting, opium for the diarrhœa, hot mustard baths for albuminuria should it occur, stimulants for collapse, etc.—*Amer. Practitioner.*

**Ether and Chloroform.**—The irresistible argument, as to the merits of the two anæsthetics, is the result of later studies of statistics. From these we learn that chloroform destroys one person out of every twenty-five hundred and four.

An analysis of the cases of deaths from ether upon which these statistics are based, show that more than one-half of them are fairly attributable to other causes than the anæsthetic, and when we consider how carelessly it is often administered, these results almost justify us in considering it an absolutely safe anæsthetic.

With chloroform, on the contrary, we are fully satisfied that no amount of care or precaution, or mode of administration, or amount inhaled, will prevent, in certain cases, the fatal result. In the light of this experience, and in the face of these facts, is any physician justified in resorting to the use of chloroform in ordinary cases? That there are exceptional cases in which it is the better agent, we admit; but we hold that the physician assumes a solemn responsibility who selects the more dangerous anæsthetic only because more convenient in some respects to give, and pleasanter for the patient to take, and thus unnecessarily subjects his patient to a risk, however small, of losing his life.—*Buffalo Med. and Surg. Journal.*

**Action of Various Diuretics.**—Dr. Maurel gives the result of his experiments (*Bulletin General de Therapeutique*) as follows:

1. Nitrate of potassium, uncertain as to the quantity of liquid, augments the solid material of the urine to a notable degree. The most active doses are a drachm to a drachm and a half.

2. Chlorate of potassium, less active with respect to the augmentation of solids, increases the fluids of the urine to a greater degree.

3. Acetate of potassium is uncertain, as to the quantity of both solids and fluids.

4. Iodide of potassium, far from being a diuretic, even seems to diminish the quantity of urine.

5. Salicylate of sodium, uncertain as to the quantity of liquid, increases the solid constituents of the urine.

6. Of three vegetable substances experimented upon—squill, colchicum and digitalis—the latter alone is a real diuretic. It augments at the same time the quantity of both solids and fluids. Dr. Maurel gives it as his opinion that no diuretic acts when the system is in a febrile condition; this must be modified before diuresis can occur.—*Buffalo Med. and Surg. Journal.*

**Magnet for the Removal of Particles of Steel and Iron from the Interior of the Eye.**—McCuen first published a notice of removal of metallic bodies in 1874, with a magnet; but Hirschberg first actually employed a magnet for such purposes. Hirschberg's apparatus was a cylinder, the ends of which were drawn out and connected with wires that formed its positive and negative poles. The amount of magnetic polarity was, however, insufficient, and, in addition, a generating aparatus was demanded. Dr. E. Gruening, of N. Y., exhibited a magnet. His idea was to have a permanent battery. His instrument consists of an armature of six parallel cylinders, magnetized, one end of the bundle of cylinders being furnished with a tapering needle, about 22 mm. long. In his first experiments, upon pigs' eyes, Dr. G. broke all the needles. Two magnets, or "magazines," were presented, the larger being less highly magnetized than the smaller one. The latter carries the weight of two steel keys and ring, about 28 grammes. The better instrument was better made by Reynders, of New York.—*Va. Med. Monthly.*

**New Operation for Prolapsus Uteri.**—A radical operation of a novel character for the relief of prolapsus uteri, has been originated by Lefort, of Paris. It consists in uniting the anterior and posterior vaginal walls along their mesial lines, so as to make two vaginas instead of one. After the operation the two vaginas lie in lateral proximity like a double-barrelled shot-gun. The surfaces freshened are about half an inch wide and two inches long, and are held in position by sutures. The operation is said to be not difficult of performance, and quite successful in preventing prolapse.—*Western Lancet.*

**Oophorectomy.**—One hundred and thirty cases of what is commonly known as Battey's operation are on record, of which Prof. A. Hegar, of Freiburg, reports forty-two. Of these one hundred and thirty, one hundred and nine have been performed by laparotomy and twenty-one by elytrotomy. The average mortality in the total number of cases has been about twenty per cent., the cases by elytrotomy showing a per centage of recoveries slightly more favorable than those from laparotomy. Although this operation may still be regarded as sub judice, the results seem to indicate that it is of value. In certain classes of cases, notably those of hystero-epilepsy, in which the prognosis without Battey's operation would be hopeless, the operation has been followed by favorable results in a sufficient number of cases to render its performance admissible.—*Chicago Med. Review.*

**Hypodermic Injections of Ergotine in Prolapsus of the Rectum.**—According to M. Vidal, in *Bulletin de l'Academie de Medecine*, prolapsus of the rectum may be readily cured, and in a comparatively short time, by hypodermic injections of ergotine. By this new method, Vidal has succeeded in curing three adult cases, of which he gives the details. He used (gr. xv) one gram of Bonjean's extract of ergot or ergotine in (ʒjss) five grams of cherry laurel water. Each injection consisted of fifteen to twenty drops (exceptionally 25), which is equivalent to twenty or twenty-five centigrams of ergotine; or, in other words, to the extract of a gram and a half to three grams of ergot. None of the injections were followed by inflammation or abscess. Bonjean's ergotine causes a rather sharp, burning pain; the solution of iron is much better tolerated. In the future the author will give preference to the latter.—*Med. Journal and Examiner.*

**Iodide of Potassium in Cough.**—Dr. John M. Shaller, in the Cincinnati Lancet and Clinic reports a case in which the administration of iodide of potassium, in combination with morphia, for the relief of troublesome cough in a phthisical patient, produced all the symptoms of a severe coryza which were succeeded, after the third dose, by violent delirium lasting about three hours. The coryza continued for forty-eight hours, finally passing away during the continued administration of the remedy.

The amount of the iodide which caused the delirium was only about twelve grains.

**For Removing Nitrate of Silver Stains,** Dr. Kraetzer recommends, in *Archives de Pharmacie*, a solution of 10 parts of corrosive sublimate dissolved in 100 parts of water. This liquid is said to remove stains from the hands, linen, wool or cotton without injuring the fabric. It is said to act much more promptly than cyanide of potassium.

**Hemorrhoids Treated with Capsicum.**—In cases of hemorrhoidal congestion, Vidal regards capsicum annum as the best remedy. He prescribes four or five pills daily, each containing 20 centigrams, half at breakfast time and half at supper time. Under this influence the congestion and all the painful symptoms which accompany it disappear rapidly.—*Journal de Medecine.*

